# Internal friction angle model of particles

**DOI:** 10.1038/s41598-022-05891-8

**Published:** 2022-02-07

**Authors:** Jiri Zegzulka, Jan Necas, Jiri Rozbroj, Daniel Gelnar, Álvaro Ramírez-Gómez, Lucie Jezerska

**Affiliations:** 1 VSB-TU Ostrava, CEET, ENET Centre, Bulk Solids Centre, 17. listopadu 15, 708 00 Ostrava, Czech Republic; 2 Department of Mining Engineering and Safety, Faculty of Mining and Geology, VSB-TU Ostrava, 17. listopadu 15, 708 00 Ostrava, Czech Republic; 3 Department of Mechanical, Chemical and Industrial Design Engineering, Universidad Politécnica de Madrid, Ronda de Valencia 3, 28012 Madrid, Spain

**Keywords:** Materials science, Coarse-grained models

## Abstract

Currently, pressure from industry to streamline processes by creating their simulation models, and thus to gradual digitization is increasing. The essence of representative simulation models of bulk materials is to understand the principles and laws of the real behavior of particles. The aim of this study is therefore to find and quantify the possibilities and principles of how particles can change their position relative to other particles. The possibilities of particle displacements were expressed using their specific trajectories and work ratios, or internal friction angle values. This created a new comprehensive model of the internal friction angle of particles independent of particle size. It enables the interpretation of the determined values of the angles of internal friction of particles and its application in the field of simulations of mass and process models. The model can be used to determine the basic composition of particles in volume and the dominant ways of their mutual displacements.

## Introduction

In the field of particulate material mechanics, it may seem that the general question of particle displacement is solved by the assumption of quasi-linear motion based on infinitesimal or at least sufficiently small particles compared to the space in which they move. The example might be shear stress versus ratio of shear cell diameter D to particle size^1^.

Shear tests to determine the parameters of friction and flow are very suitable methods for describing the properties of particulate materials^2–5^. Shear machine manufacturers use different shear cell designs and, based on size, also recommend different ratios of maximum particle size to the characteristic size of these cells^6,7^.

In Jenike's direct shear test, the shear plane is not ideally horizontal^1,6^. The actual shear direction deviates angularly from the imaginary horizontal shear plane. It is more of a shear zone than a plane. Particle size and normal loading have a significant effect on the properties of the shear zone. Numerous experiments were performed on Jenike's shear test, where the shape of the shear zone was demonstrated, for example, by X-ray scanning^8^.

The current state of particle research allows for more detailed studies of particle behavior using discrete element simulations (DEM). Lots of works have focused on efficiently determining the optimal particle shape for simulation processes^9,10^. These process methods are validated according to the volumetric behaviors of the materials. There is a direct correlation with the effect of particle shape properties on volumetric and strength behavior together with the change in internal friction angle^11^. To evaluate the complex material properties based on internal friction, the effect of particle shape on internal friction can be included.

Shear tests have been the subject of much research focusing on DEM^12^. The diversity of force distribution, particle directions and velocities, and the effect of particle size on the shear zone, its shape and size were also demonstrated using DEM simulations^13,14^. The results of experiments and simulations show that the shear zone is not a horizontal plane and its shape is demonstrably related to particle displacements.

An ideal shear plane would be created by precise shear (cross-section) of particles in a shear cell, or by the shear of infinitesimal particles.

Dilatancy in granular materials is another important concept^15^. Dilatation here means a change in volume which is caused by quasi-static shear deformation. Reynolds stated that the angle of friction used by Rankin is a macroscopic quantity “Related to the arrangement of particles”^16^. It has been proven that friction between particles is much less important in determining the strength of granular materials in macro-dimensions than their “arrangement”^16–18^.

The very essence of the continuum of dry crystalline materials applied to the principles of shear cell and particle size ratios can be further understood as the number of possibilities of changing the position of particles in a volume element (space) relative to each other.

If it were shown that one particle has a limited number of possibilities to change its position relative to other particles, then the total number of changes in the position of all particles will also be quantifiable (final).

This paper describes a new perspective on the internal friction angle of particles. The proposed model is based on a non-zero particle size with a symmetric shape that is embeddable in a sphere (basic shapes).

Historically, there are some tendencies to simplify the interpretation of internal friction through an angle of repose, e.g.^19^. This study focuses on the partial effects of particle shifts, which can occur as macro properties of matter (angle of repose, etc.).

## Internal friction angle model of particles

Mechanical work is given by the scalar product of force and path. In particle mechanics, it is the product of the external force acting on the particle and the magnitude of its displacement. In general, much attention has always been paid to questions of force interactions between particles, while minimal attention has been paid to the question of determining the possible trajectories of particles as their position relative to the environment changes. The fact is that for the determination of mechanical work, both force and displacement affect the resulting value in the scalar product of force and trajectory.

The infinitesimal increment of mechanical work is given by the scalar product *d****W*** (1), where ***F*** is the force acting on the particle and *d****s*** is an infinitesimal displacement vector along the trajectory of the particle. Equation (2) then applies to three-dimensional vectors.

1 $$d{\varvec{W}} = {\varvec{F}}\cdot d{\varvec{s}}$$

2 $$dW = F_{x} \cdot dx + F_{y} \cdot dy + F_{z} \cdot dz$$

### General definition of the internal friction angle

The general definition of internal friction is based on energy balance which describes the ability of particles of matter to do work.

**Figure 1:**
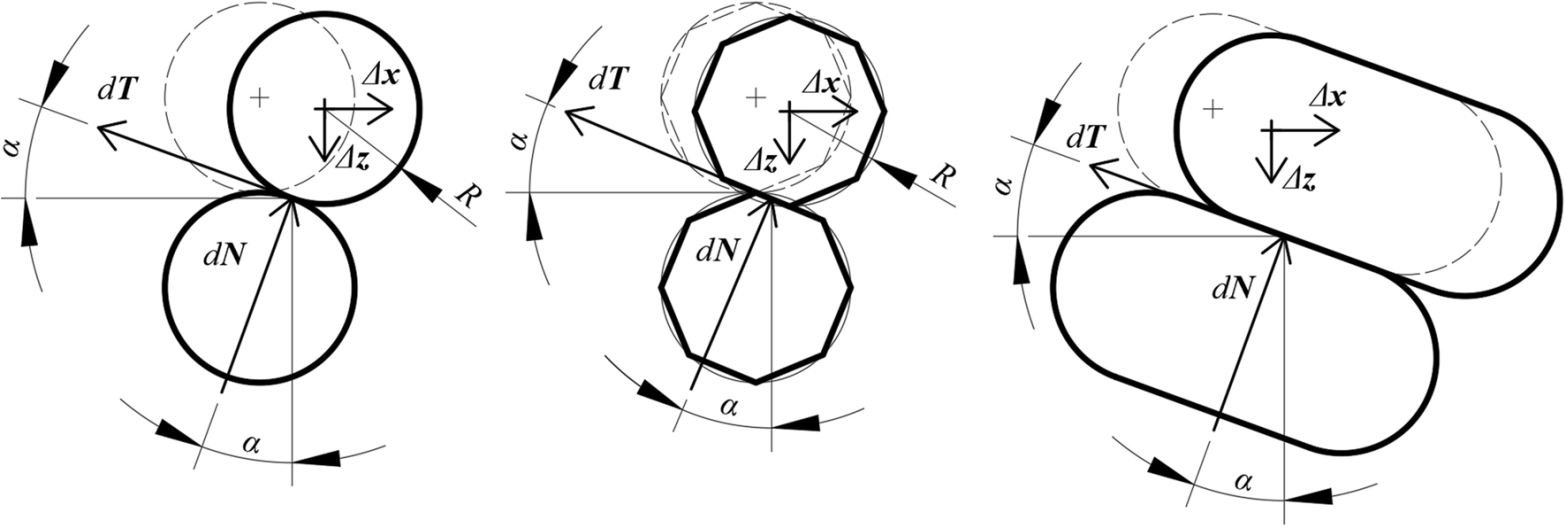
Diagram of the work of particles during displacement.

The situation in Fig. 1 assumes dry (Coulomb) friction without actual rotation of bodies where the kinetic friction force *T* is equal to the product of the kinetic friction coefficient *tan*(*φ*) and the normal force *N* and its direction is opposite to the slip direction. The path of bodies (particles) is given by their shape.

The ratio of the works *dW1* and *dW2* can be considered as a generalized tangent of the angle of internal friction and this ratio can be written in the following form (see Eq. 3):

3 $$\tan (\varphi ) = \frac{{dW_{1} }}{{dW_{2} }} = \frac{{\left\| {d{\varvec{T}}} \right\| \cdot \left\| {\Delta {\varvec{x}}} \right\| \cdot \cos (\alpha )}}{{\left\| {d{\varvec{N}}} \right\| \cdot \left\| {\Delta {\varvec{z}}} \right\| \cdot \cos (\alpha )}} = \frac{{d{\varvec{T}} \cdot \Delta {\varvec{x}}}}{{d{\varvec{N}} \cdot \Delta {\varvec{z}}}}$$

where *α* is the angle between the vectors *d****T*** and *Δ****x*** or the vectors *d****N*** and *Δ****z***. The basic model assumes that the force vectors are parallel to the displacement vectors if the angle *α* is zero or approaches the limit of zero (Eq. 4).

4 $$\mathop {\lim }\limits_{\alpha \to 0} (\cos (\alpha )) = \cos (0) = 1$$

Then, the ratio of force magnitudes will be equal to parameter *B*

5 $$B = \frac{{\left\| {d{\varvec{T}}} \right\|}}{{\left\| {d{\varvec{N}}} \right\|}}$$

and the ratio of the size of the displacement will be equal to parameter *C*.

6 $$C = \frac{{\left\| {\Delta {\varvec{x}}} \right\|}}{{\left\| {\Delta {\varvec{z}}} \right\|}}$$

Solving the ratio defined in Eq. (3) leads to three possible interpretations of friction which are given by the conditions:

- Small or symmetrical displacements and greater friction forces (Eq. 5)
- Larger displacements and smaller magnitude friction forces (Eq. 6)
- Combination of both (Eqs. 5 and 6)

#### Small or symmetrical displacements and greater magnitude friction forces

If we do not know the ratio of the displacement lengths of the particles *C*, we can solve the situation by assuming very small (infinitesimal) particles (with a characteristic radius *R* → 0). When their displacements ǀǀ*Δx*ǀǀ and ǀǀ*Δz*ǀǀ are infinitesimally small, the task can be defined by the limit of the ratio of the respective displacements (Eq. 7). The influence of small particle size is the subject of papers dealing with the determination of boundary conditions for measurability of samples on shear machines^1,20,21^.

7 $$\mathop {\lim }\limits_{{\left\| {\Delta x} \right\| \to 0 \wedge \left\| {\Delta z} \right\| \to 0}} (C) = 1$$

The characteristic radius *R* represents the maximum grain size. Ideally, the shape is symmetrical and spherical, but in the real world it is made up of infinitely many surfaces. For simpler graphical representation in this paper, the real shape of the particles formed by asymmetrical surfaces will be replaced by a spherical shape.

The parameter that depends on the characteristic particle size (on the characteristic radius *R*) is parameter *C* (Eq. 6). It expresses the influence of the geometric parameters of the particles on the value of the internal friction angle.

For the limiting case of particle size approaching zero, we can write the existential condition of parameter *C* (displacements ǀǀ*Δ****x***ǀǀ and ǀǀ*Δ****z***ǀǀ are a function of particle size with characteristic radius *R*), or have particles such that *R* → 0 ⇒ ǀǀ*Δ****x***ǀǀ → 0 ∧ ǀǀ*Δ****z***ǀǀ → 0. If we are dealing with lengths of displacement vectors ǀǀ*Δ****x***ǀǀ and ǀǀ*Δ****z***ǀǀ approaching zero, we can afford to assume that their ratio is equal to 1.

It follows from this that for a particle size of zero, the dissipative work ratio in Eq. (3) is not a function of the displacement vectors, but a ratio of the magnitudes of the forces. The tangent of the internal friction angle (Eq. 8) is given by the product of the ratio of the magnitudes of the forces *B* (Eq. 5) and the ratio of the magnitude of the displacement *C* = 1 (Eq. 6).

8 $$\tan (\varphi ) = \frac{{\left\| {d{\varvec{T}}} \right\|}}{{\left\| {d{\varvec{N}}} \right\|}}$$

**Figure 2:**
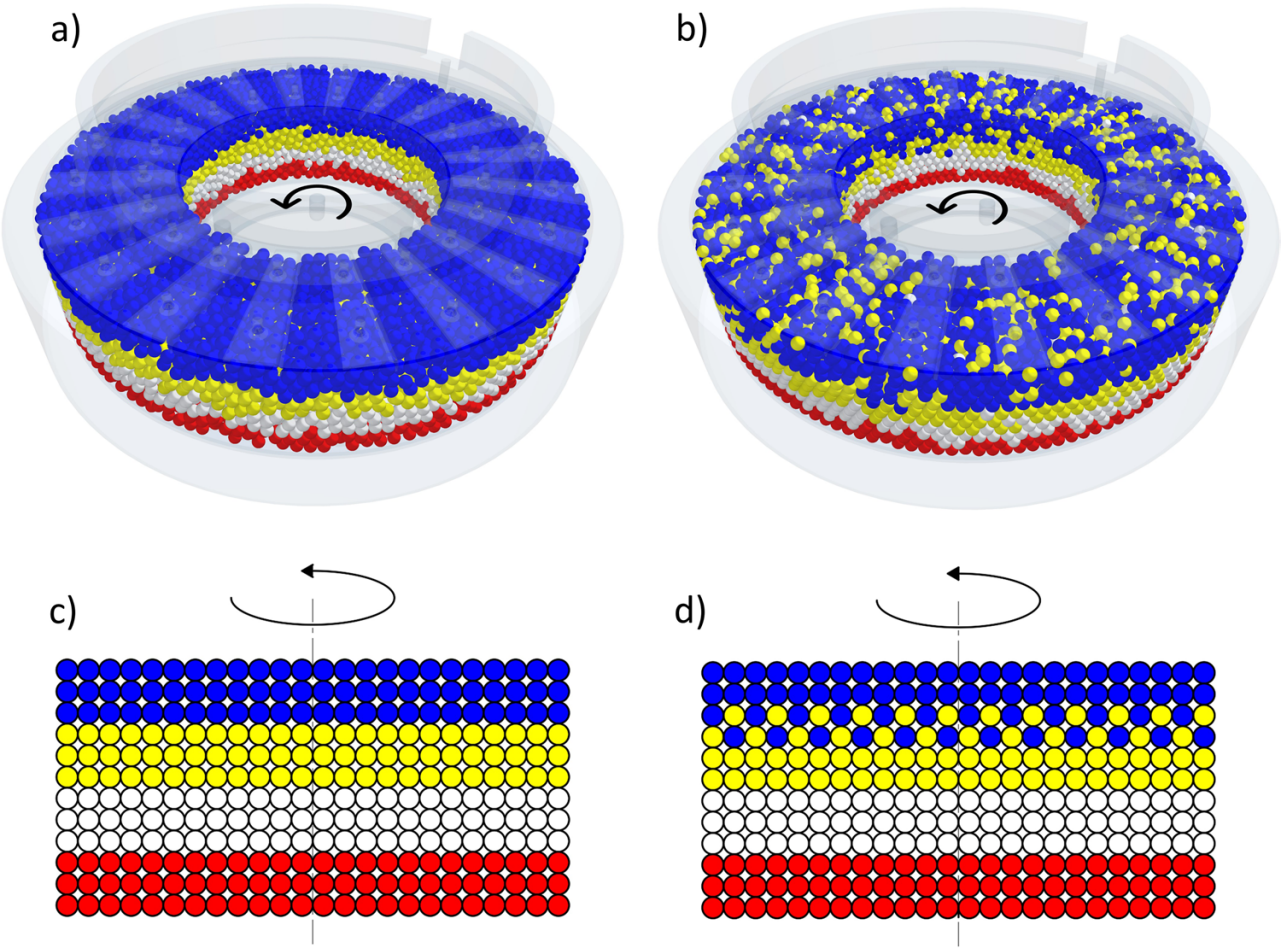
Particle displacement. (**a**) Initial position of the rotary test, (**b**) final position of the rotary test, (**c**) schematic representation of the initial position, (**d**) schematic representation of the displacement.

Under ideal conditions and for non-zero symmetrical particle sizes (without deformations, particle degradation and moisture), particle displacements occur in the shear cell while maintaining the sample volume. If the volume is constant, it is possible that there is a finite number of possible particle displacements limited by duration (time). In the case of a rotary shear test, there is no path constraint dictated by geometry and the basic displacements may repeat in cycles (Fig. 2).

**Figure 3:**
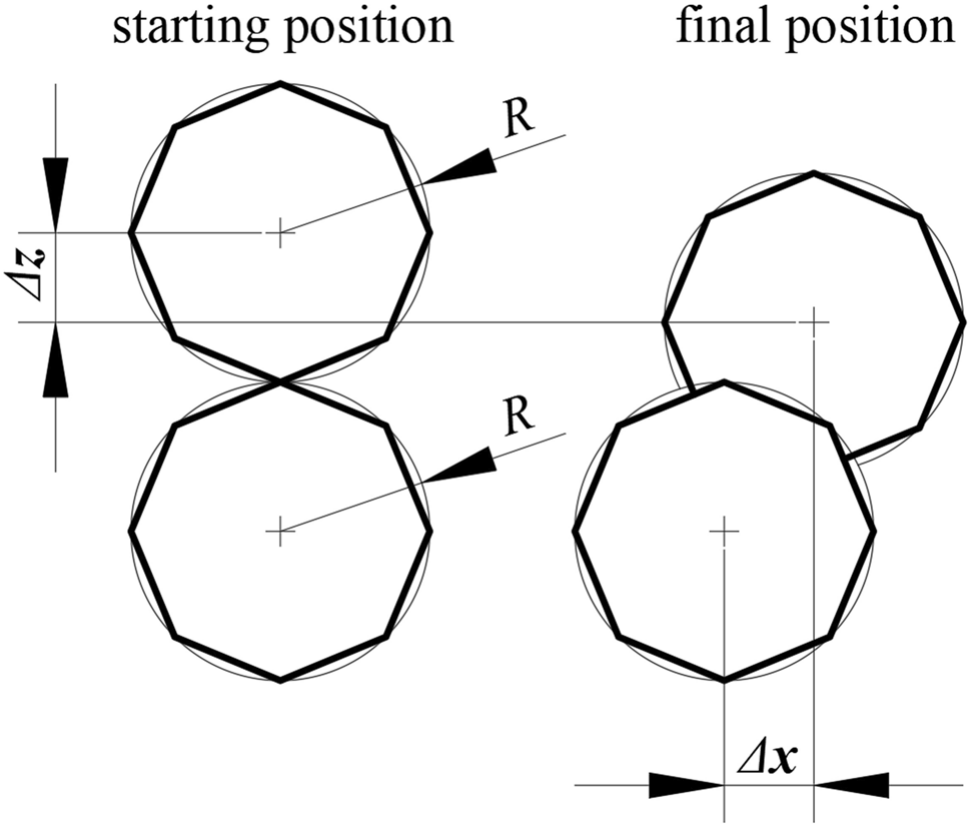
Possibility of symmetrical values of displacements ǀǀ*Δ****x***ǀǀ and ǀǀ*Δ****z***ǀǀ.

Equation (8) can also assume that ǀǀ*Δ****x***ǀǀ = ǀǀ*Δ****z***ǀǀ even if the ratio of lengths *C* would be 1. This situation is explained in Fig. 3. The upper particle is in contact with the lower and shifts by ǀǀ*Δ****x***ǀǀ = ǀǀ*Δ****z***ǀǀ, or both particles can shift by the same values of ǀǀ*Δ****x***ǀǀ and ǀǀ*Δ****z***ǀǀ.

Assuming a particle size of zero or ǀǀ*Δ****x***ǀǀ = ǀǀ*Δ****z***ǀǀ, we can also write Eq. (9) for the shear stress and Eq. (10) for the normal stress. It can also be assumed that the shear surface *Aτ* is equal to the normal surface *Aσ*, i.e., *Aτ* = *Aσ* = *A*, and therefore the tangent of the inner angle of friction is usually written as Eq. (11).

9 $$\tau = \frac{dT}{{dA}} = \frac{{\left\| {d{\varvec{T}}} \right\|}}{{A_{\tau } }}$$

10 $$\sigma = \frac{dN}{{dA}} = \frac{{\left\| {d{\varvec{N}}} \right\|}}{{A_{\sigma } }}$$

11 $$tan(\varphi ) = \frac{\tau }{\sigma }$$

#### Small magnitude friction forces, large displacements

Assuming that the magnitude of the forces performing the work approaches zero, we can solve Eq. (3) analogously based on the limit of the ratio of the magnitude of the forces *B* (see Eq. 5) with the condition of the magnitude of the forces approaching zero. Particles of matter are only displaced by external forces in the environment (affecting other particles). Particles of matter move, for example, by passing through the gaps between particles. Particles fall through and friction fluctuates (transitions between static and kinetic friction, or slip-stick effect) due to the unevenness of the surfaces formed by the particles when the particles move amongst each other.

If we are dealing with the magnitudes of vector forces ǀǀ*d****T***ǀǀ and ǀǀ*d****N***ǀǀ approaching zero, or if the internal friction angle *φ* → 0 ⇒ ǀǀ*d****T***ǀǀ ≈ ǀǀ*d****N***ǀǀ (perfect fluid/inviscid fluid), we can afford to introduce a similar assumption as used in section “Small or symmetrical displacements and greater magnitude friction forces”, namely that the ratio of the magnitude of the forces *B* is equal to 1, or

12 $$\mathop {\lim }\limits_{{\left\| {d{\varvec{T}}} \right\| \to 0 \wedge \left\| {d{\varvec{N}}} \right\| \to 0}} (B) = 1$$

It follows that the tangent of the angle of internal friction is given by the product of the ratio of the path lengths *C* and the ratio of the magnitude of the forces equal to 1, or

13 $$tan(\varphi ) = \frac{{\left\| {\Delta {\varvec{x}}} \right\|}}{{\left\| {\Delta {\varvec{z}}} \right\|}}$$

Equation (13) represents a situation where the magnitude of the force ratio *B* is negligible with respect to the magnitude of the path ratio *C*. The internal friction angle of the particles is then defined independently of the force effect and is dependent on the displacement of the particles for particle systems.

#### Combination of small or symmetrical displacements with greater frictional force and small frictional forces with large displacements

Proportional dissipative work can be expressed by the tangent of an angle *φ*:

14 $$tan(\varphi ) = B \cdot C$$

where both parameters are nonzero. The solution is complicated because both the ratio of force magnitudes and the ratio of displacement lengths are complex functions of many physical quantities and the solution is subject to the definition of complicated contact tasks, the solvability of which is still determined by the degree of optimization of mathematical models in calculating specific solutions.

## Overview of particle displacement possibilities

**Figure 4:**
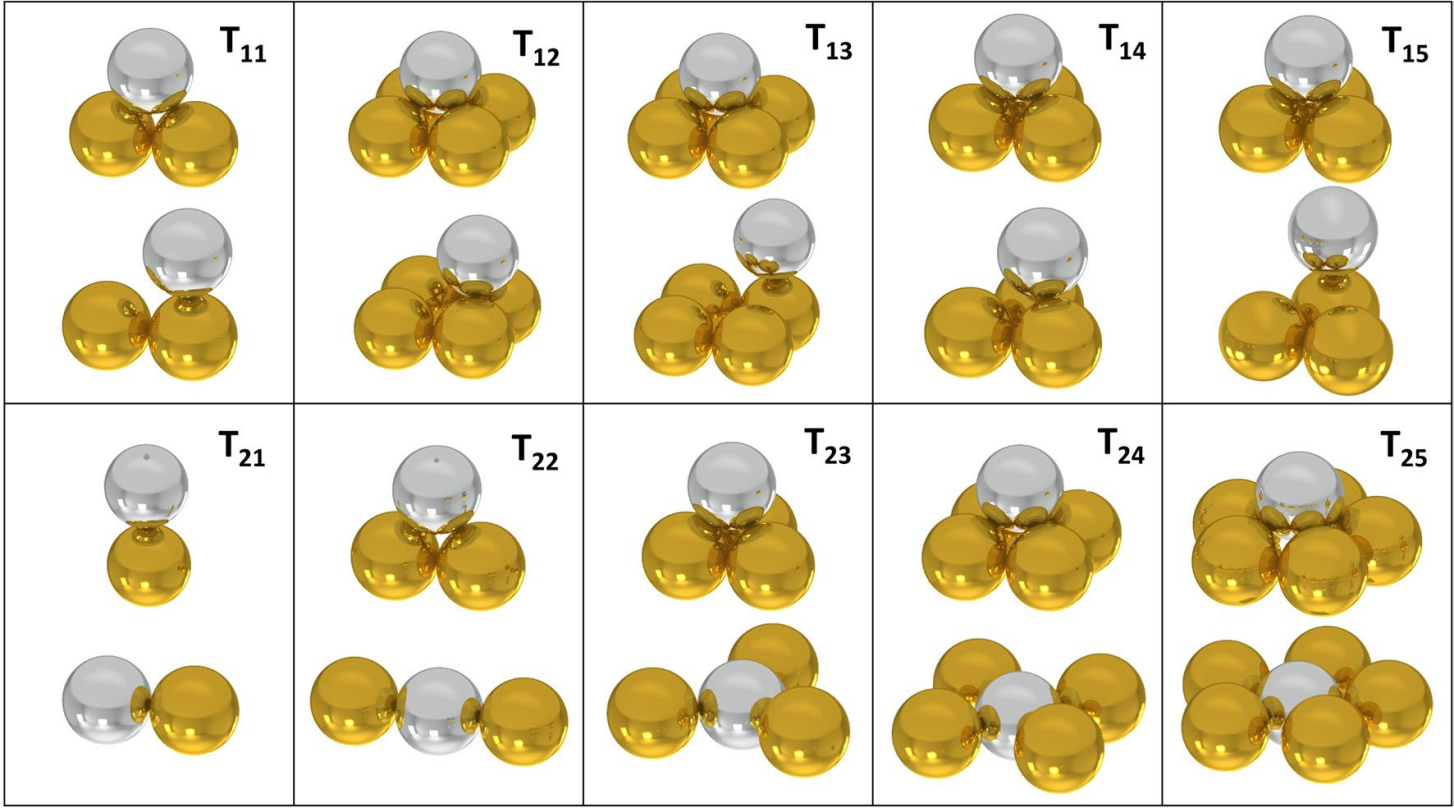
Initial and terminal positions of the displacements T_11_–T_15_ and T_21_–T_25_.

The model of internal friction of particles is based on the basic shape contacts of particles and the differences in distance between the particles of these shape contacts. The first group T_11_–T_15_ (Fig. 4) is characterized by the fact that the active particle “goes around” the passive particle^22^. The second group T_21_–T_25_ (Fig. 4) is characterized in that the active particles displace passive particles. Figure 4 shows the initial and final position of the particle of the individual particle movements.

The value of *Δz* represents the maximum possible path of the particle in the vertical direction and also the height difference of the position of the particle. The calculation was performed as the difference between the maximum and minimum height values for the displacements T_11_–T_15_ that the spherical contour of the particle can perform (Eq. 15). For T_21_–T_25_ shifts, the value of *Δz* is directly equal to the maximum height (Eq. 16). The value of *Δx* represents the displacement of the particle in the horizontal direction so that the maximum value of *Δz* is always attained. Each displacement is specific in its own combination of *Δz*/*Δx* values and is independent of the particle radius *R* parameter (Table 1). Table 2 then shows the work ratios *dW1* and *dW2*, or value *tan*(*φ*) and internal friction angle of particles *φ*.

15 $$\Delta z = H_{max} - H_{min}$$

16 $$\Delta z = H_{max}$$

**Table 1:** Parameters *Δz* and *Δx* of individual displacements T_11_–T_15_ and T_21_–T_25_.

	*Δz*	*Δx*
T11	$2 \cdot R - R \cdot tg\;(60^\circ )$	$R$
T12	$R \cdot tg(60^\circ ) - \sqrt {(R \cdot tg(60^\circ ))^{2} - R^{2} }$	$R$
T13	$2 \cdot R - \sqrt {(R \cdot tg(60^\circ ))^{2} - R^{2} }$	$\sqrt {R^{2} + R^{2} }$
T14	$R \cdot tg\;(60^\circ ) - \frac{2 \cdot R}{3} \cdot \sqrt 6$	$\frac{R \cdot tg(60^\circ )}{3}$
T15	$\frac{2 \cdot R \cdot sin(60^\circ )}{3} \cdot 2$	$\frac{2 \cdot R \cdot sin\;(60^\circ )}{3} \cdot 2$
T21	$2 \cdot R$	$2 \cdot R$
T22	$R \cdot tg\;(60^\circ )$	$R$
T23	$\frac{2 \cdot R}{3} \cdot \sqrt 6$	$2 \cdot R - \sqrt {(2 \cdot R)^{2} - \left( {\frac{2 \cdot R}{3} \cdot \sqrt 6 } \right)^{2} }$
T24	$\sqrt {(R \cdot tg(60^\circ ))^{2} - R^{2} }$	$2 \cdot R - \sqrt {(2 \cdot R)^{2} - (R \cdot tg(60^\circ ))^{2} - R^{2} }$
T25	$\sqrt {\frac{{\left( {2 \cdot 2 \cdot R} \right)^{2} - \left( {\frac{2 \cdot R}{{\sin \left( \frac{180}{5} \right)}}} \right)^{2} }}{4}}$	$2 \cdot R - \sqrt {\left( {2 \cdot R} \right)^{2} - \frac{{\left( {2 \cdot 2 \cdot R} \right)^{2} - \left( {\frac{2 \cdot R}{{\sin \left( \frac{180}{5} \right)}}} \right)^{2} }}{4}}$

**Table 2:** Summing up of *tan*(*φ*) and *φ* of individual displacements T_11_–T_15_ a T_21_–T_25_.

	T_11_	T_12_	T_13_	T_14_	T_15_	T_21_	T_22_	T_23_	T_24_	T_25_
*tan*(*φ*)	0.268	0.318	0.414	0.172	0.318	1.000	1.732	1.932	2.414	3.520
*φ*, °	15.0	17.6	22.5	9.7	17.6	45.0	60.0	62.6	67.5	74.1

## Mean probable angle of internal friction of particles

With the same probability of attainment of the number *n* of individual displacements, the mean probable angle of internal friction of particles *φ*c can be expressed by Eq. (17). The coefficient *k*_Tij_ represents the probability of individual displacement T_11_–T_15_ and T_21_–T_25_. In our model case, *k*_T11_–*k*_T25_ equals the value 1 and after achieving *φ*c = 39.2°.

17 $$\varphi_{c} = \frac{{\sum k_{{T_{ij} }} \cdot \varphi_{{T_{ij} }} }}{n}$$

## Experimental measurement of the internal friction angle of crystalline material

**Figure 5:**
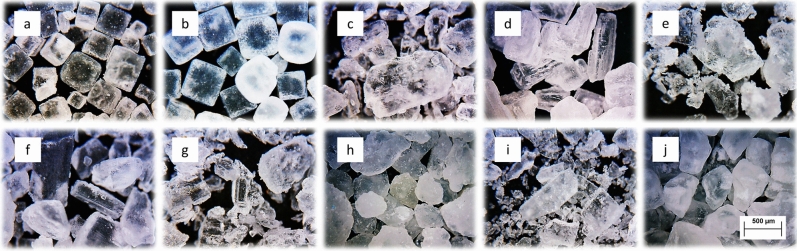
View of the grains of the measured salt samples, (**a**) edible iodized salt, (**b**) pure natural salt, (**c**) fine sea salt, (**d**) coarse-grained sea salt, (**e**) Sicilian fine salt, (**f**) dehydrated sea salt, (**g**) fine coastal sea salt, (**h**) Sicilian coarse-grained salt, (**i**) edible stone salt, (**j**) Italian coarse-grained sea salt.

Due to its stable crystallization in a cubic system and the possibility of inserting a crystal shape into a sphere, a dry crystalline material in the form of NaCl salt was chosen (Fig. 5). Particle size distribution was measured on devices Camsizer Retch and Cillas 1190. The Table 3 shows the measured particle size values. The designation of the salt samples is the same as in Fig. 5.

**Table 3:** Particle size distribution of salt samples.

Salt samples	*d10 *(µm)	*d50* (µm)	*d90* (µm)	*Span S *(–)
a	373	524	708	0.6
b	442	585	746	0.5
c	314	770	1077	1.0
d	1609	3031	4555	1.0
e	84	253	430	1.4
f	224	474	806	1.2
g	52	824	1202	1.4
h	1198	1999	3031	0.9
i	382	725	1176	1.1
j	1492	2115	3379	0.9

The measurement of internal friction was performed on a Ring Shear Tester RST-01.pc. The normal load for Pre-Shear was set at 20, 10, 5 kPa. Individual normal loads were measured ten times. The lowest measured value of the normal load for shear was set to 10% of the normal load for Pre-Shear and the number of stress levels was 6.

The angle of internal friction at steady-state flow *φsf* was averaged both for the partial normal load for Pre-Shear (20, 10, 5 kPa) and for all three of these stresses of each salt sample. The resulting value of *φ*sfC was then arrived at by averaging all values of *φ*sf. This angle characterizes the internal friction at steady state flow in the section plane (friction bulk solid / bulk solid)^3,7^. Table 4 summarizes the measured *φ*sf values.

**Table 4:** Average values of φsf for 20, 10, 5 kPa and overall average values of salt samples a-j.

	a	b	c	d	e	f	g	h	i	j
20 kPa	35.8	34.7	37.4	38.3	39.2	38.7	40.4	40.3	40.1	40.2
10 kPa	34.1	35.0	36.8	38.8	37.5	38.0	38.8	39.7	39.6	40.1
5 kPa	32.4	34.2	35.3	36.8	36.7	37.0	37.6	39.2	38.5	39.6
Average	34.1	34.6	36.5	38.0	37.8	37.9	38.9	39.8	39.4	39.9

**Figure 6:**
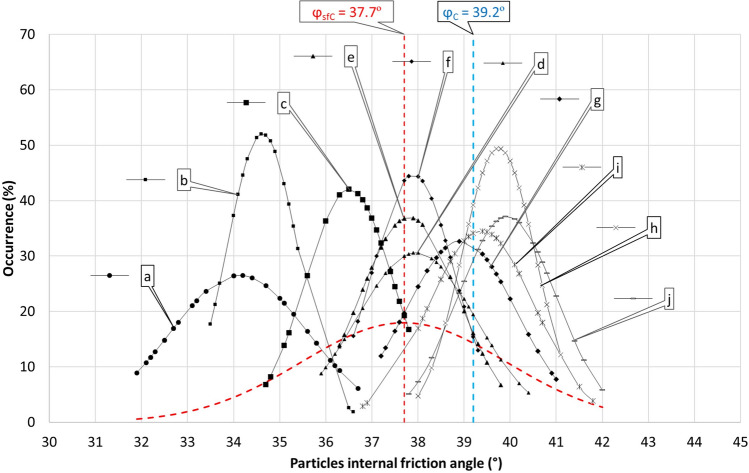
Gaussian distribution *φ*sf for individual samples, for the whole set of samples *φ*sfC and mean probable angle of internal friction of particles *φ*C.

Figure 6 shows an overview of experimental *φ*sf data processed into a Gaussian distribution for individual salt samples, but also as a whole as for one set of *φ*sfC salt samples. Furthermore, the derived mean probable angle of internal friction of particles *φ*C is indicated here. Since there is no perfect overlap of these two values, it can be concluded that the probability of individual displacements under consideration is not uniform or the same as considered in the model, but tends to a certain imbalance.

## Conclusion

The paper introduces the principle of describing the internal friction of particles using a probabilistic model of particle shape displacements. A relationship was found between the model of shape angles of internal friction of particles and experimentally determined angle of internal friction of particles at steady flow. The formation of particle displacement and the balance of internal friction are based on changes in the positions of the particles relative to the environmental changes in their positions.

The nature of the motion of both individual particles and their sets within the body of particulate matter implies that the achievement of motion is conditional upon the motion autonomy of individual particles and their clusters. The autonomy of motion of individual particles makes it possible to characterize the flow capabilities of non-cohesive bulk materials.

The model presented in this paper is based on the description of the properties of the motion of matter:

- particles can change their position based on shape contacts which define the conditions of their movement
- the way in which particles change their position is the dominant factor characterizing matter in terms of dissipation work needed to achieve these movements
- the way in which the particles change their position determines the energy intensity of the mass system and thus the size of the angle of internal friction of the particles
- with the same probability of all particle displacements, the mean probable angle of internal friction of the particles is 39.2°.

The presented model enables both the interpretation of the measured values of the internal friction angle and the application of the measured values in the field of simulations of mass models and mechanical processes.

The trajectory of moving particles of particulate matter may not always be directly dependent on external forces exerting mechanical work. Internal friction can be understood as a measure of loss work and the angle of internal friction as a ratio of loss work. The work performed, i.e., the scalar product of the external force and the particle trajectory, is the product of two independent quantities. The external force is a function of the external inputs and the trajectories are a function of the position of the particles (particle configuration) before motion and changes in their positions during motion.

Active particles generally have two ways in which they can change their position relative to the surrounding particles. The first way is that the active particles do not push the passive particles out of their positions, but move around them. The second method is that the active particles push the passive particles out of their positions and occupy the original position of the passive particles.
